# Porcine Deltacoronavirus (PDCoV): From Emerging Swine Enteric Coronavirus to a Potential Coronavirus to Infect Humans

**DOI:** 10.3390/v18101055

**Published:** 2026-09-23

**Authors:** Xiaorong Chen, Zhishui Zhang, Hanyu Cheng, Siyuan Qi, Pengfei Wang

**Affiliations:** Shanghai Pudong Hospital, Fudan University Pudong Medical Center, Shanghai Institute of Infectious Disease and Biosecurity, State Key Laboratory of Genetics and Development of Complex Phenotypes, School of Life Sciences, Fudan University, Shanghai 200438, China; 25110700008@m.fudan.edu.cn (X.C.); 24307110087@m.fudan.edu.cn (Z.Z.); 24307110029@m.fudan.edu.cn (H.C.); 24307110032@m.fudan.edu.cn (S.Q.)

**Keywords:** porcine deltacoronavirus, biological characteristics, epidemiology, pathogenic mechanisms, antibody development

## Abstract

The global dissemination of coronaviruses continues to pose formidable threats to public health and socioeconomic development, making these viruses a major research priority. Porcine deltacoronavirus (PDCoV), an emerging porcine enteric virus identified over the past two decades, has resulted in substantial economic losses to the swine industry owing to its high mortality and persistent circulation across the globe. Beyond swine, PDCoV possesses a broad host spectrum and is capable of infecting avian species, mice, calves, and other animals. Most alarmingly, its zoonotic potential was highlighted in 2021 through the discovery of PDCoV genomic RNA in pediatric samples from Haiti, highlighting its potential to become the eighth coronavirus species known to infect humans. Despite these dual veterinary and public health threats, licensed vaccines and specific human therapeutics remain unavailable. These facts highlight an urgent demand for comprehensive and in-depth studies on PDCoV to mitigate its potential risks and forestall future outbreaks. This comprehensive review summarizes the latest research advances in PDCoV in four aspects: biological characteristics, epidemiology, pathogenic mechanisms, and antibody development. It is expected that this review can provide a robust theoretical basis for understanding PDCoV infection dynamics and accelerating the development of next-generation, broad-spectrum antiviral strategies to mitigate future interspecies spillover events.

## 1. Introduction

Porcine deltacoronavirus (PDCoV) is an enveloped, single-stranded, positive-sense RNA virus belonging to the order *Nidovirales*, family *Coronaviridae*, subfamily *Orthocoronavirinae*, and genus *Deltacoronavirus* [1,2,3]. Alongside the alphacoronaviruses porcine epidemic diarrhea virus (PEDV) and transmissible gastroenteritis virus (TGEV), PDCoV (a deltacoronavirus) is a significant enteric pathogen primarily causing diarrhea, vomiting, and dehydration in piglets, with co-infections commonly observed in clinical settings [4]. While its natural host is primarily swine, the virus demonstrates a broad host range, capable of cross-species infection in calves, chickens, turkeys, and mice [5,6,7,8]. Detection in serum samples from Haitian children further highlights its zoonotic potential [9]. This comprehensive review summarizes the current research on PDCoV from biological characteristics, epidemiology, pathogenesis, and antibody development, aiming to provide a theoretical basis for its prevention and control.

## 2. Biological Characteristics

### 2.1. Virion Morphology and Structure

The PDCoV virion is roughly spherical, pleomorphic, with a diameter of approximately 60–180 nm [1,10,11]. The outer layer of the viral particle is a lipid bilayer envelope embedded with three structural proteins, forming the characteristic crown-like spikes. Among these, the spike (S) protein forms prominent rod-like trimers and is the key protein mediating receptor binding and membrane fusion with host cells [12]. The membrane (M) protein is a triple-spanning transmembrane protein that plays a central role in virion assembly and morphogenesis; its C-terminal endodomain participates in interactions with other viral proteins [13]. The Envelope (E) protein, present in smaller quantities, is involved in virion release and the modulation of host immune responses [14]. The interior of the virus contains a helically symmetric nucleocapsid formed by the genomic RNA tightly coiled with the nucleocapsid (N) protein [15,16] (Figure 1a).

### 2.2. PDCoV Genomics

#### 2.2.1. Evolutionary Lineages

PDCoV exhibits high genetic variability, driven by two primary mechanisms: point mutations and genetic recombination [17,18,19]. Point mutations are predominantly enriched in the S gene, which evolves at a faster rate than other viral genes and is under positive selection [19]. In contrast, recombination events occur mainly in the ORF1ab gene, where most recombination breakpoints are located [19]. The identification of natural recombinant strains, such as BN23, further demonstrates that PDCoV recombination contributes to its adaptive evolution during transmission, and may also enhance its virulence and expand its host range [17]. Several nomenclature systems have been proposed to describe PDCoV phylogeny. Initial phylogenetic investigations grouped strains into three major geographic lineages—China, North America/USA, and Southeast Asia [20,21]. Subsequent whole-genome analyses uncovered additional phylogenetic substructure within these geographic groups, particularly among Chinese and Southeast Asian viruses [18]. Most recently, a hierarchical framework comprising two major lineages and four sublineages (1.1, 1.2, 2.1, and 2.2) has been introduced, providing a more refined representation of PDCoV evolutionary relationships. This classification is based on comprehensive genomic data available in GenBank, allowing for a more robust and globally representative phylogenetic framework than earlier geographically restricted studies [22].

The China lineage mainly comprises isolates from mainland China and Hong Kong. The USA lineage is more widespread, including isolates from the United States, Mexico, South Korea, and others. Notably, strains belonging to the USA lineage have also been isolated in some parts of China, suggesting that transcontinental pork trade may facilitate the cross-border spread of the virus [23], potentially leading to genetic exchange between different lineages. The Southeast Asia lineage is primarily distributed in Thailand, Vietnam, and other Southeast Asian countries, as well as southern China.

The China lineage exhibits high substitution and recombination rates, indicating strong mutational potential [18]. Its internal genetic diversity provides a rich gene pool for recombination. The recombination rate of the USA lineage is relatively lower, suggesting it may rely more on fine point mutations at key sites in the S protein to gain transmission advantages [19,24]. The Southeast Asia lineage has numerous branches, a complex evolutionary process, and high genetic diversity, associated with multiple recombination events with PDCoV strains from other lineages [18]. A characteristic molecular marker of this lineage is a continuous nucleotide deletion in the *Nsp2/Nsp3* region of the *ORF1a* gene [25].

#### 2.2.2. Genomic Structure

The PDCoV genome is a single-stranded, positive-sense RNA approximately 25 kb in length, making it one of the smaller genomes among known coronaviruses (Figure 1b). Its 5′ end possesses a cap structure, and its 3′ end has a poly(A) tail, structurally resembling eukaryotic mRNA, enabling direct translation upon entering host cells [1,3].

The genomic order (from 5′ to 3′) is as follows:

Replicase genes (*ORF1a*/*ORF1b*) occupy about two-thirds of the genome, encoding two large polyproteins, pp1a and pp1ab. These are cleaved by viral proteases to produce 15 nonstructural proteins (nsp2–nsp16) involved in viral transcription and translation [1,3,26,27,28,29,30]. Some of these nsps constitute the viral replication–transcription complex (RTC), responsible for viral RNA synthesis and processing, while others participate in suppressing host immune responses and regulating the translation of both host and viral proteins, creating a favorable environment for viral replication [24,25]. Fehr et al. [30] provide a comprehensive summary of coronavirus nsp functions. Nevertheless, investigations into PDCoV non-structural proteins remain incomplete, and their exact biological functions have yet to be systematically delineated.

The *S* gene encodes the S protein, a type I transmembrane glycoprotein forming trimers on the viral surface. During infection, the S protein is cleaved by host proteases into two functional subunits: S1, responsible for recognizing and binding host cell receptors (e.g., porcine aminopeptidase N, pAPN; human aminopeptidase N, hAPN) [7,31]; and S2, which mediates virus–host cell membrane fusion and is associated with cytopathic effects, viral replication, assembly, and release [25]. In most coronaviruses, the receptor-binding domain (RBD) is located in the C-terminal domain of the S1 subunit (S1-CTD). For PDCoV, S1-CTD constitutes an immunodominant region likely containing major neutralization epitopes. The S protein is the primary target of neutralizing antibodies and a key antigen for vaccine development.

The *E* gene encodes the E protein, the smallest structural protein of PDCoV. Its structure consists of three parts: a hydrophilic N-terminus, a hydrophobic transmembrane domain (TMD), and a hydrophilic C-terminus. The TMD forms an amphipathic α-helix, the key structural basis for its ion channel activity. The E protein assembles into homopentamers in the membrane, forming an ion channel-type viroporin. It interacts with the M protein to drive virion formation and release. Its ion channel is selectively permeable to ions like Na^+^, K^+^, and Cl^−^, with channel activity linked to processes such as viral release, inflammasome activation, and apoptosis. Through its C-terminus, the E protein interacts with host proteins, disrupting tight junctions and inducing inflammatory cytokine release; its ion channel activity can activate the NLRP3 inflammasome, exacerbating inflammatory responses. Additionally, the E protein can induce the unfolded protein response (UPR) and apoptosis, regulating the balance between viral replication and host cell death [14,32].

The *M* gene encodes the M protein, the most abundant structural protein in the viral envelope. The M protein exists as a dimer and is a triple-spanning transmembrane protein with three transmembrane domains; its N-terminus is located externally (can be glycosylated), and its C-terminus is located internally. The C-terminus of the M protein has a long endodomain involved in interactions with other viral proteins [13]. Through interactions with the S, N, and E proteins, the M protein drives virion assembly; it can also stabilize the nucleocapsid structure via interaction with the C-terminus of the N protein [13]. The conformation of the M protein is closely related to its function: it exists in two conformational states—MLONG and MCOMPACT. The MLONG conformation binds to the viral ribonucleoprotein (RNP) complex, inducing membrane curvature and promoting the formation of spherical particles; the MCOMPACT conformation is associated with membrane relaxation and particle elongation [13].

The *NS6* gene is an accessory gene encoding the non-structural protein 6 (NS6) protein. Research indicates that NS6 is crucial for efficient PDCoV replication both in vivo and in vitro. It primarily localizes to the endoplasmic reticulum (ER) and the ER-Golgi intermediate compartment (ERGIC), suggesting a potential role in viral assembly [33]. Furthermore, NS6 has been confirmed as a type I interferon (IFN-β) antagonist: it interacts with host intracellular retinoic acid-inducible gene I (RIG-I) and melanoma differentiation-associated protein 5 (MDA5), interfering with their ability to recognize viral dsRNA, thereby inhibiting IFN-β production and aiding viral immune evasion [33].

The *N* gene encodes the N protein, a highly phosphorylated RNA-binding protein that complexes with the viral genomic RNA to form a helical RNP complex [15,16]. Like other coronaviruses, the N protein contains two main domains: an N-terminal domain (NTD) and a C-terminal domain (CTD), connected by a variable linker region [15]. The N protein was shown to undergo self-assembly via non-covalent oligomerization and, through its RNA-binding activity, protects viral RNA from degradation and participates in virion assembly [16]. It also acts as an interferon antagonist, suppressing host innate immune responses, and may influence cell survival and death through interactions with host proteins [34]. Due to its high abundance and strong immunogenicity, the N protein is often used as an antigen for the serological detection of PDCoV infection [34].

The *NS7* gene is located within the *N* gene [24] and, like NS6, is an accessory gene. The specific function of NS7 remains unclear and requires further study [35,36,37].

### 2.3. Life Cycle

#### 2.3.1. Cell Entry

PDCoV primarily enters host cells via its S protein binding to receptors on the cell surface. It has been confirmed that pAPN is a functional receptor for PDCoV [7,31]. Following S protein–receptor binding, the virus can enter host cells via different pathways: one involves direct membrane fusion at the cell surface after receptor binding; the other involves entry via endocytosis, with membrane fusion occurring subsequently within endosomes [38]. Research shows that various proteases (e.g., trypsin, cathepsins) can influence the selectivity and efficiency of these two entry pathways (Figure 2) [38].

#### 2.3.2. Replication, Transcription, and Translation

After viral RNA is released into the cytoplasm, the ORF1a/ORF1b region at the 5′ end is first recognized and translated by the host cell into two large polyproteins, which are then cleaved into multiple non-structural proteins [11,25]. The synthesized non-structural proteins migrate to regions like the ER, where the ER membrane remodels to form characteristic membrane structures such as double-membrane vesicles (DMVs) and zippered endoplasmic reticulum. The non-structural proteins assemble on these membranes to form the viral RTC [11,39]. The precise molecular mechanisms of this membrane rearrangement are not yet fully understood [11,39]. Once the RTC is formed, the virus uses the genomic positive-strand RNA as a template to synthesize negative-strand RNA, which in turn serves as a template for synthesizing full-length progeny genomic RNA and a set of nested subgenomic mRNAs (sg mRNAs) [40]. The mRNAs for viral structural proteins are translated into their corresponding proteins by host cell ribosomes on irregularly enlarged rough endoplasmic reticulum (rER) (Figure 2) [11].

**Figure 2:**
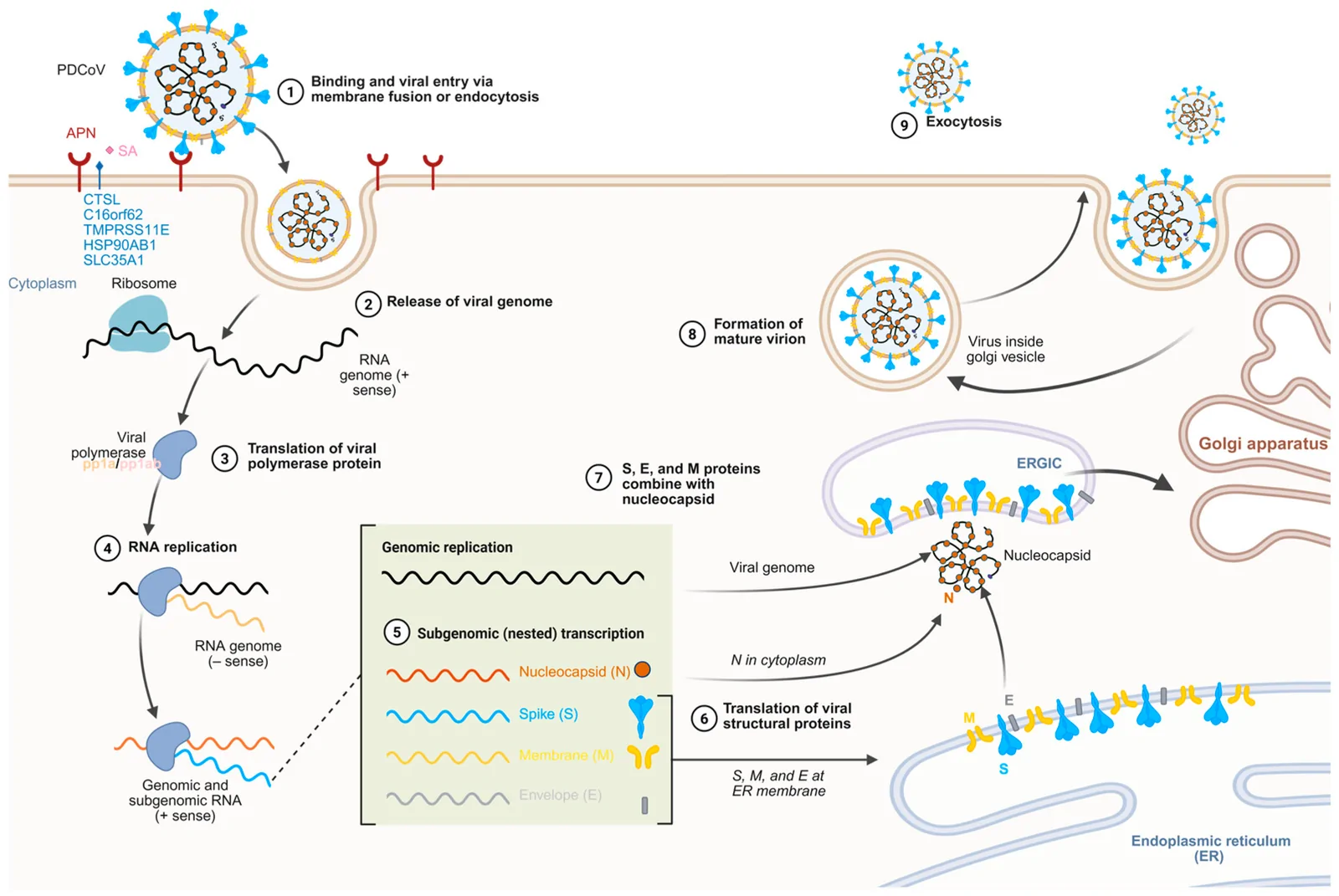
Schematic representation of the PDCoV replication cycle in host cells. PDCoV propagation operates through a tightly coordinated cascade of cellular events, encompassing viral entry, intracellular replication and transcription, virion morphogenesis, and progeny release. Several host factors—including C16orf62 [41], SLC35A1 [42], HSP90AB1 [43], CTSL and TMPRSS11E [44]—have been shown to positively modulate PDCoV infection, putatively by influencing receptor availability or components of the membrane fusion apparatus. However, direct physical interactions between these host proteins and the viral S protein have not yet been experimentally demonstrated. Abbreviations: DMV, double-membrane vesicle; ER, endoplasmic reticulum; ERGIC, ER–Golgi intermediate compartment; rER, rough endoplasmic reticulum; RTC, replication–transcription complex; sg mRNA, subgenomic messenger RNA; pAPN, porcine aminopeptidase N; CTSL, cathepsin L; TMPRSS11E, transmembrane serine protease 11E; S, spike protein; E, envelope protein; M, membrane protein; N, nucleocapsid protein. https://BioRender.com/e5rtou3, accessed on 15 September 2026.

#### 2.3.3. Assembly and Release

The progeny viral genomic RNA combines with abundantly synthesized N protein to form the nucleocapsid [34]. Subsequently, PDCoV virions assemble in the expanded rER as immature precursors with an electron-dense periphery and clear core. These precursors are then trafficked through the ER-Golgi intermediate compartment (ERGIC) to the Golgi complex, where they complete structural maturation into infectious virions (Figure 2) [11]. During this process, PDCoV, similar to other coronaviruses, may induce autophagy in cells, but the specific mechanisms remain incompletely elucidated and require further exploration [11]. Mature viral particles accumulate within secretory vesicles, which often merge to form larger vesicles. These vesicles containing mature virions are transported to the cell periphery, where they ultimately fuse with the plasma membrane, releasing the virions extracellularly via exocytosis or cell lysis, thereby infecting new host cells [11].

## 3. Epidemiology

### 3.1. Global Distribution

PDCoV was first discovered in Hong Kong, China, in 2012 [1]. The first PDCoV strain (HKU15) was detected via molecular surveillance of coronavirus diversity in rectal swabs from healthy pigs [1]. The virus gained major international attention in early 2014 when severe viral diarrhea broke out in swine herds across Ohio, US [45]. Within months, it spread across at least 18 US states and into Canada. Retrospective serological testing using an S1-based ELISA detected anti-PDCoV IgG antibodies in archived serum samples dating back to 2010 [46]. To monitor the rapid escalation of Swine Enteric Coronavirus Disease (SECD), the United States Department of Agriculture (USDA) issued a Federal Order in June 2014 designating PDCoV and PEDV as mandatory reportable pathogens. Over the four-year official monitoring period preceding the mandate’s rescission in March 2018, federal surveillance logged 300 confirmed and 88 presumed positive PDCoV premises [47]. Phylogenetic analysis based on all complete PDCoV genome sequences suggests the virus has been present in mainland China for at least 20 years, with a retrospective study confirming its presence in diarrheic pigs in Anhui province, China, as early as 2004 [48]. PDCoV is now distributed across Asia and the Americas, while remaining absent in several other continents.

Since its first report in mainland China in 2015, PDCoV has rapidly spread within pig populations nationwide. According to a 2023 study, the pooled prevalence of PDCoV infection in Chinese pig populations is estimated at 12.2% (95% CI: 10.2–14.2%). Regional prevalence was highest in Central China (24.5%, 95% CI: 16.1–32.9%), North China (18.5%, 95% CI: 9.7–27.3%), and South China (12.2%, 95% CI: 9.0–15.3%). In contrast, Northeast and Northwest China had lower PDCoV positivity rates of 3.9% (95% CI: 2.4–5.3%) and 3.1% (95% CI: 1.1–5.2%), respectively. From 2014 to 2021, the estimated prevalence of PDCoV infection peaked in 2015 at 20.5% (95% CI: 10.1–31.0%) and was lowest in 2021 at 4.8% (95% CI: 2.3–7.3%), showing an overall declining trend. PDCoV infection rates in sows and suckling piglets were 23.6% (95% CI: 15.8–31.4%) and 20.4% (95% CI: 11.5–29.4%), respectively, significantly higher than in weaned and growing-finishing pigs [49]. Since January 2015, 244 PDCoV outbreaks have been reported across 22 production systems and 186 sites in 16 U.S. states. Historical incidence peaked in December 2018 (18 cases across 4 systems) and February 2021 (20 cases across 3 systems). Similarly, the 2023 peak occurred in February, recording 13 cases across 3 production systems [50]. This substantial baseline prevalence underscores the widespread nature of the virus across the world.

The origin of PDCoV remains unclear. After years of effort, researchers have identified other members of the Deltacoronavirus genus in birds, leading to the hypothesis that birds are the natural host for deltacoronaviruses. Genomic studies of PDCoV show a high degree of correlation between the PDCoV genome and the avian coronavirus HKU17 [51]. Subsequent research suggests that the sparrow coronavirus HKU17 and coronavirus HKU11 may have recombined to generate PDCoV [51]. Transmission between animals, possibly involving unknown species, may promote genetic recombination and lead to the virus spreading between different species.

### 3.2. Host Range

#### 3.2.1. Poultry

PDCoV possesses potential for cross-species transmission, and several non-porcine species have shown susceptibility to infection under experimental conditions (Figure 3). Studies indicate that multiple avian species, including chickens, ducks, geese, sparrows, and pigeons, are susceptible to PDCoV infection [6]. While intra-flock transmission is limited in chickens, it is highly efficient within pigeon populations. A survey of coronavirus infection in Chinese poultry showed that among 5249 avian samples from 17 provinces in China, the PDCoV positivity rate in pigeon samples was 23.14% (159/687), significantly higher than in duck samples (0.55%, 8/1451), geese (0.20%, 1/494), and chickens (0.08%, 2/2567) [52]. Studies on PDCoV in sparrow populations show that infected sparrows appear clinically healthy, and PDCoV-like virus in sparrow fecal samples produced only a faint band on agarose gel after RT-PCR, suggesting inefficient replication in this host [53]. These data imply that PDCoV is primarily prevalent in pigeons, although avian species may serve as potential susceptible hosts and could contribute to virus spillover under certain conditions. Furthermore, the observed transmission of PDCoV to co-housed sentinel birds [54] highlights the possibility that putative avian hosts may facilitate the virus’s ability to cross the avian–mammalian species barrier.

#### 3.2.2. Mammals

Studies on mammalian hosts show that ferrets, leopard cats, mice, and calves can be infected with PDCoV under experimental conditions. Research on ferrets showed limited serological changes and tissue damage post-infection, with no clinical symptoms. However, the detection of high viral RNA loads in ferret intestinal tissues and prolonged fecal virus shedding suggests ferrets may warrant further investigation as potential intermediate hosts for PDCoV cross-species transmission [55]. Inoculation experiments in mice showed that BALB/c and Kunming mice are susceptible to PDCoV, but no obvious clinical symptoms were observed in infected mice, except for weight loss in inoculated BALB/c mice. Histopathological examination revealed that the intestinal walls of PDCoV-inoculated BALB/c mice became transparent and thin, with accumulation of yellowish fluid in the intestinal lumen and obvious pulmonary hemorrhages. These lesions gradually recovered as the disease progressed. Examination of viral distribution in mice showed high levels of viral RNA in the kidneys and intestines, and mice developed an antibody response [56]. In calves, oral inoculation with PDCoV resulted in acute infection characterized by the absence of diarrhea, other clinical symptoms, or histopathological lesions; however, viral RNA was detected in feces, and an immune response was observed [5].

#### 3.2.3. Humans

Evidencing its evolutionary plasticity, PDCoV has demonstrated the capacity to cross the species barrier into humans. In a surveillance initiative beginning in May 2014, plasma samples were collected from Haitian children presenting to school clinics with acute undifferentiated febrile illness. Among 369 samples collected through December 2015, three children (aged 5–10 years) were identified as infected with coronavirus strains directly clustering with PDCoV. All three patients presented with fever and subsequently recovered without complications. Phylogenetic analysis revealed that these human isolates did not originate from a single introduction event; specifically, strains from cases 1 and 3 formed a well-supported monophyletic clade with Chinese swine lineages, whereas the case 2 strain clustered independently alongside US swine strains [9]. Although PDCoV infection in these children was mild—representing an acute febrile positivity rate below 1% and suggesting a limited immediate public health threat—cross-sectional studies inherently detect only acute, symptomatic viremia. Therefore, broader serological investigations across diverse populations are required to determine true infection frequencies, despite diagnostic challenges posed by cross-reactivity with endemic human coronaviruses. Nonetheless, these empirical findings underscore the potential for PDCoV zoonotic spillover, particularly in rural or underdeveloped regions with frequent livestock contact [9].

Synthesizing these findings, one possible evolutionary hypothesis is that PDCoV may have originated in wild birds, then crossed species to sparrows, then to mammals, and finally to humans via cross-species transmission. This hypothesized evolutionary scenario is largely supported by accumulating genomic evidence from diverse deltacoronaviruses circulating among avian hosts, though direct field-derived evidence documenting each sequential host-jump event remains sparse. While current confirmed human cases are limited to viral RNA detection in Haitian pediatric patients and lack evidence of sustained human-to-human transmission, the broad experimental host tropism of PDCoV still raises public-health concerns regarding its spillover risk at the animal–human interface. Given this broad host range and its zoonotic potential, characterizing host lineages and maintaining active surveillance across domestic and wild mammalian populations are important for global public health security. Such continuous monitoring will help detect emerging spillover events early and provide fundamental data for developing targeted prevention and intervention strategies against future PDCoV-related threats.

### 3.3. Infection Symptoms

#### 3.3.1. Clinical Symptoms

The typical clinical symptoms of PDCoV infection in pigs are acute and severe watery diarrhea, vomiting, and dehydration, sometimes accompanied by lethargy and anorexia. Clinical symptoms generally appear 1–2 days post-inoculation, with diarrhea progression most severe at 3–7 days post-infection (dpi), followed by gradual recovery [57]. The overall infection rate ranges from 19.62% to 29.19%, with mortality in newborn piglets around 40% [58], and up to 80% for the CHN-ANHZ-2024 strain [59]. PDCoV-associated disease has been reproduced in gnotobiotic (GN) pigs. According to an inoculation experiment with three GN pigs: watery diarrhea occurred suddenly, severely, and persistently in all three experimental piglets within 20 h post-oral inoculation. Vomiting was observed in piglets infected for 48 h and 72 h, but core body temperature remained normal. No respiratory signs (cough and nasal discharge) were observed. Despite diarrhea and progressive dehydration, appetite was maintained. The most lethargic and severely ill piglet died 24 h post-infection, one died at 48 h, and the third at 72 h. The overall findings in the three inoculated GN piglets were similar: flanks and hind legs were covered with yellow adherent diarrheic feces; the small intestine was dilated, filled with gas and fluid, with thin and translucent walls; the stomach was filled with curdled milk; and the cecum, spiral colon, and distal colon were dilated and filled with yellow intestinal fluid [60].

#### 3.3.2. Histopathological Symptoms

Histopathological studies showed that the duodenum, jejunum, and ileum from infected pigs exhibited mild to severe villous atrophy, consistent with viral infection. Lesions varied significantly among pigs, primarily showing diffuse villous blunting, fusion, and attenuation of enterocytes in the mid-to-distal jejunum and ileum [61]; the duodenum and proximal jejunum were less affected or unaffected. Lesions included acute diffuse, severe atrophic enteritis and mild hydropic degeneration of superficial epithelial cells in the cecum and colon [62]. At 4 dpi, some pigs showed mild attenuation or severe flattening and necrosis of villous enterocytes, with degenerated enterocytes sloughing into the lumen. Additionally, a small number of lymphocytes and neutrophils infiltrated the moderately contracted lamina propria, occasionally with apoptotic debris and vascular congestion. Similar lesions were observed at 7 dpi, including mild to moderate attenuation of enterocytes and necrosis with sloughing, with low numbers of neutrophils and lymphocytes in the lamina propria [27]. Gastric epithelial cell degeneration and necrosis, increased fundic gland curvature, and gastric pit dilation were also observed [57].

#### 3.3.3. Comparison with Other Porcine Enteric Viral Infections

The overall clinical disease induced by PDCoV infection is very similar to that caused by TGEV and PEDV, except that vomiting is more common in PDCoV-infected piglets, and mortality is lower (approximately 40% to 50%) compared to PEDV and TGEV (90% to 100%). Similar to TGEV and PEDV, PDCoV replicates extensively in the small intestine. High levels of genomic RNA are detected in feces, intestinal contents, and tissues. Numerous antigen-positive cells are detected in all regions of the small intestine. The intestinal histopathology of PDCoV-infected piglets resembles that seen with PEDV and TGEV infections [62]. Finally, moderate levels of PDCoV RNA are detected in the blood and extra-intestinal tissues, similar to the transmission characteristics of TGEV and PEDV infection in piglets. PDCoV has two unique infection features distinguishing it from TGEV and PEDV infection. First, PDCoV infection causes epithelial lesions in gastric pits, whereas gastric mucosal epithelial involvement has not been reported for TGEV or PEDV. A report documenting gastric lesions associated with CoV RNA in convalescent human SARS patients sets a precedent for this finding [63]. Second, PDCoV infection is accompanied by mild interstitial pneumonia. Small amounts of PDCoV RNA were found in lung homogenate cells, and PDCoV antigen was detected in bronchial mucosal epithelial cells. Pulmonary pathological changes have not been reported for PEDV or TGEV [57].

#### 3.3.4. Infection Symptoms in Other Species

As mentioned, calves, chickens, mice, and other animals are susceptible to PDCoV infection. PDCoV-inoculated chickens developed diarrhea, shed detectable viral RNA from the cloaca and trachea, and had gastrointestinal distension with yellow fluid and gas mixtures. Histological lesions were observed in the lung, kidney, and intestinal tissues of infected chickens [6,54]. PDCoV-inoculated calves shed viral RNA for a long duration but showed no clinical symptoms or obvious intestinal lesions, indicating limited infectivity of PDCoV in calves [5]. PDCoV can also infect mice to a limited extent. PDCoV RNA was mainly detected in the intestines and lymphoid tissues of mice, with no similar symptoms observed [56]. As mentioned before, Haitian children in whose plasma samples PDCoV RNA was detected exhibited mild clinical symptoms, including fever, cough, and abdominal pain, but recovered spontaneously [9]. Such asymptomatic or mild infections suggest that these hosts may be potential susceptible species for PDCoV and could potentially contribute to interspecies transmission, but the field epidemiological evidence is still lacking.

### 3.4. Transmission Routes

#### 3.4.1. Direct Transmission

Direct transmission of PDCoV occurs through contact with various secretions (feces, vomit, saliva, nasal discharge, oral fluids) from infected pigs. Testing of 293 pig samples from the US and Canada showed PDCoV positivity in 52% (27/52) of intestinal samples, 40% (30/75) of fecal samples, 32% (6/19) of feed samples, 17% (15/90) of fecal swabs, 50% (1/2) of vomit samples, 0% (0/1) of environmental samples and 19% (10/54) of saliva samples [45]. Contamination from different sources reflects the complexity of potential transmission. The fecal–oral route is considered the most important pathway for PDCoV transmission. Feces and vomit from infected pigs contain high concentrations of the virus, often persisting for weeks, and fecal shedding may continue even after clinical symptoms completely resolve [64].

#### 3.4.2. Indirect Transmission

Indirect transmission occurs via virus-contaminated feed, feed trucks, transport trailers, and personnel. Contaminated feed and transport trailers may play an important role in virus spread within and between farms. In a study on PEDV transmission, 6.6% (38/575) of trailers at six processing plants were contaminated before unloading, and 5.2% (28/537) of trailers not contaminated upon arrival became contaminated during unloading [65]. Similar to PEDV, this is likely an important transmission route for PDCoV, but direct evidence is limited. Another study on viral survival in swine feed showed PDCoV could persist for up to 42.04 days in soybean meal and 25.60 days in corn [66]. Therefore, contaminated feed can also serve as an indirect transmission route.

#### 3.4.3. Airborne Transmission

Aerosolized PEDV can be transmitted via air over long distances downwind, and the potential for airborne transmission of PDCoV should also be considered. A controlled experiment showed that when healthy pigs were physically separated from PDCoV-infected pigs by permeable material, the healthy pigs developed diarrhea within 3 to 4 days, and PDCoV was detected in them [67]. Potential aerosol or other airborne transmission routes could significantly enhance PDCoV infectivity and warrant further investigation.

## 4. Pathogenic Mechanisms

### 4.1. Intestinal Injury

PDCoV is an enterotropic coronavirus. Its pathogenesis begins with exposure in the intestinal lumen and proceeds through receptor- or attachment factor-mediated entry, intracellular replication, and epithelial injury, accompanied by antagonism of innate immunity and the subsequent induction of adaptive immunity. Current evidence indicates that PDCoV replicates efficiently in villous enterocytes of the small intestine, particularly in the jejunum and ileum, causing villous atrophy, reduced absorptive surface area, and watery diarrhea. Viral RNA or antigen can also be detected in extraintestinal tissues, but the presence of viral nucleic acid should not be equated with productive replication or a direct pathogenic role without supporting evidence [57,58]. Besides intestinal changes, mild multifocal broncho-bronchiolocentric nonsuppurative interstitial pneumonia was identified in the lungs 48 h post-infection, and ascites, hydrothorax, and thymic atrophy were detected in the piglet at 72 h. Clinical manifestations were essentially similar across infections with different strains [10]. In cell culture, one PDCoV replication cycle takes approximately 5–6 h, and viral genomic RNA and infectious titers usually peak around 24 h post-infection, providing a kinetic basis for rapid epithelial damage [57,58].

At the tissue level, the major target cells are villous epithelial cells in the small intestine. Enterocyte injury, sloughing, and villous atrophy reduce absorptive capacity and compromise barrier integrity, and these changes have been directly documented in PDCoV-infected pigs [46,47]. Beyond this absorptive deficit, PDCoV also directly disrupts the intestinal mucosal barrier by downregulating tight-junction components (ZO-1, claudin-1, and claudin-5) and, through activation of the Notch signaling pathway, inhibiting intestinal stem cell differentiation into goblet cells and reducing MUC-2 mucin secretion [68]. This goblet-cell loss and mucus depletion further compromise barrier function and contribute to diarrhea [68]. The resulting osmotic imbalance and increased fluid secretion are widely accepted to drive diarrhea, but this latter cascade has been characterized most thoroughly for PEDV and TGEV and is inferred for PDCoV by analogy rather than directly measured [57,58,69,70]. Because strain, dose, age, and sampling time strongly influence pathological readouts, cytopathic effects or apoptosis observed in a particular cell line should not be directly generalized to all in vivo intestinal lesions [57,58,70].

### 4.2. Receptor Recognition and Cross-Species Infection

The best-supported protein receptor or entry cofactor for PDCoV is APN/CD13. Early studies showed that the PDCoV S1B domain interacts with APN, and ectopic pAPN expression can confer PDCoV binding, entry, and productive infection in otherwise poorly permissive cells, supporting pAPN as a functional entry receptor [57,71]. Structural work further resolved PDCoV RBD complexes with hAPN and pAPN, showing that the RBD contacts conserved regions in APN domains II and IV. In hAPN, residues such as Y316, K379, E426, and W429 form a hydrogen-bond and salt-bridge network that is important for viral binding and infection [71].

PDCoV can use APN orthologues from different species in experimental systems, including porcine, human, and avian APN, and these findings help explain its broad cell-entry capacity [57,71]. However, APN-knockout pigs remain susceptible to PDCoV, and CD163/pAPN double-knockout pigs show reduced susceptibility rather than complete resistance [57,71]. Thus, the most rigorous wording is that APN/CD13 is the most clearly characterized PDCoV protein receptor or entry cofactor, but APN binding alone does not determine host range, natural transmission, or sustained zoonotic spread.

Further research in this direction is critical for elucidating the cross-species transmission potential and pathogenic mechanisms of PDCoV. A functional study showed that cholesterol depletion from host cells and viral particles using methyl-β-cyclodextrin (MβCD) markedly impaired PDCoV infectivity, whereas exogenous cholesterol supplementation partially restored it [72]. These findings indicate that cholesterol is an essential host factor for PDCoV entry. However, the potential cytotoxicity of such cholesterol-depleting reagents toward host cell membranes warrants caution in experimental settings. Yuan et al. reported that sialic acid (SA) serves as a key modulator of PDCoV infectivity [73]. As a trypsin-dependent attachment receptor, SA facilitates PDCoV adhesion to the host cell surface. Independently, Liu et al. [8] found that removal of cell-surface SA by neuraminidase (NA) treatment, as well as blockade of the S1 and S1A subunits with specific antisera, efficiently inhibited PDCoV-induced hemagglutination of human, porcine, and chicken erythrocytes. It further supports the interaction between SA and the S1A of the S protein. Nevertheless, the S1A-targeted neutralizing antibody 22C10 fails to block PDCoV-mediated hemagglutination [74], possibly owing to the spatial distance between the SA-binding pocket and the 22C10 epitope within the S1A domain. Collectively, these studies highlight the pivotal roles of cholesterol and SA in PDCoV entry. However, the precise molecular mechanisms remain poorly understood and controversial. Direct biochemical evidence for SA binding to the S protein is still lacking.

In addition, multiple host cellular factors involved in PDCoV infection have been newly identified, including Chromosome 16 open reading frame 62 (C16orf62) [41], solute carrier family 35 member A1 (SLC35A1) [42], heat shock protein 90 alpha family class B member 1 (HSP90AB1) [43], cathepsin L (CTSL) and transmembrane serine protease 11E (TMPRSS11E) [44]. These host factors have been proposed to facilitate PDCoV infection by modulating the expression of the key viral receptor APN and triggering viral–host membrane fusion. Nevertheless, the detailed molecular mechanisms underlying their regulatory functions remain poorly elucidated and incompletely understood. Notably, direct experimental evidence supporting their physical binding interactions with S protein is still lacking.

Collectively, the cellular entry of PDCoV is no longer confined to a single receptor-dependent pattern. Instead, it constitutes a sophisticated, multifactorial coordinated process that integrates functional receptors, attachment factors, the membrane lipid microenvironment, host regulatory proteins, and proteases. This complex entry machinery endows PDCoV with strong adaptability to diverse host cells and augments its pathogenic and cross-species transmission competence, which fundamentally underpins its zoonotic spillover potential.

### 4.3. Spike Activation, Entry Routes, and Replication Regulation

PDCoV entry depends on conformational rearrangement and proteolytic activation of the S protein. In the prefusion trimer, the RBD is mainly captured in a ‘lying’ state; receptor engagement likely requires transition toward a more accessible ‘standing’ state [57,71]. At least two entry routes have been described: a cell-surface protease-mediated route in which trypsin activates S cleavage and membrane fusion, and a low-pH endosomal route facilitated by cathepsins L/B [57]. Depending on the cell type, PDCoV can also exploit clathrin-mediated endocytosis, macropinocytosis, or caveolae-associated processes, indicating that entry pathways are context-dependent [57].

After entry, PDCoV manipulates host signaling to promote its own replication. ERK1/2, JNK1/2, and p38 MAPK pathways have each been linked to PDCoV replication or progeny release. ERK activation mainly affects post-entry steps, including viral RNA synthesis, protein translation, and progeny production, whereas JNK1/2 and p38 can affect viral biosynthesis, release, and cytokine expression [57,70]. PDCoV also induces autophagy, and p38-mediated autophagy can promote viral replication in some models; however, the key viral inducer of this process remains incompletely defined [57,70]. PDCoV may trigger caspase-9/caspase-3-dependent mitochondrial apoptosis in vitro to facilitate replication, but the extent of apoptosis in infected intestinal enterocytes in vivo remains debated and should be stated with appropriate qualification [57,70].

### 4.4. Immune Evasion, Barrier Breach, and Adaptive Immune Activation

For PDCoV, intestinal epithelial cells are active antiviral sensors rather than passive targets. PDCoV infection upregulates RIG-I-like receptor (RLR) signaling pathway genes and induces type I (IFN-α/β), type III (IFN-λ1), and inflammatory cytokines in vitro and in vivo [46]. Consistent with this, multiple PDCoV proteins have been shown to target components of the RIG-I/MDA5–MAVS–TBK1/IKKε–IRF3/IRF7–NF-κB axis, indicating that this signaling cascade is engaged during PDCoV infection; however, the step-by-step activation of each component has in most cases been inferred from viral protein antagonism studies rather than directly measured [46,59]. Because the IFN-λ receptor is enriched on epithelial surfaces, type III IFN is thought to be particularly relevant for antiviral defense at the gut mucosa; however, this concept has been established mainly in rotavirus, norovirus, and reovirus models, and its direct functional importance in PDCoV infection remains to be formally demonstrated [69]. PDCoV weakens this barrier through multiple viral proteins. nsp5 cleaves NEMO to inhibit IFN-β induction and cleaves STAT2 to block JAK-STAT signaling; nsp15 interferes with NF-κB nuclear translocation; NS6 has been reported to reduce dsRNA recognition by RIG-I/MDA5, although deletion of NS6 from a PDCoV infectious clone did not measurably affect its IFN-antagonizing activity, so the contribution of NS6 to IFN evasion in the context of authentic infection remains debated; NS7a competes with TRAF3 and IRF3 for IKKε binding; and the N protein suppresses RIG-I K63-linked ubiquitination and promotes porcine IRF7 degradation through the ubiquitin–proteasome pathway [57,58,69,70,75]. Moreover, it interacts with the helicase domain and C-terminal domain of RIG-I, impairing the binding of dsRNA and the protein kinase R activator (PACT) to RIG-I [76]. PDCoV infection can also reduce peroxisome abundance, thereby impairing MAVS-IRF1-associated IFN-λ1 production [57,69,70].

Immune evasion does not abolish host immunity; it mainly buys the virus time to replicate before adaptive responses mature. As epithelial injury, antigen release, and inflammatory mediators accumulate, dendritic cells and macrophages are presumed to capture viral antigens and migrate to mesenteric lymph nodes, initiating B- and T-cell responses in a manner analogous to other enteric viral infections. Humoral immunity is expected to involve mucosal sIgA and serum IgG, with neutralizing antibodies mainly targeting the S protein/RBD, and cellular immunity may involve MHC I/II-mediated activation of CD8+ cytotoxic T cells and CD4+ helper T cells, which support antibody production and clearance of infected cells through cytokines and cytotoxic mechanisms [57,58,69,70]. PDCoV-specific T-cell epitopes, long-term mucosal memory, and vaccine-induced cellular immunity are less well characterized than receptor usage and innate immune evasion.

## 5. Antibody Development

PDCoV is characterized by a high evolutionary rate, frequent genetic recombination, and substantial genetic variability [19,77,78]. These features collectively increase the likelihood that emerging variants will evade immune protection induced by early-generation vaccines. Consequently, the development of vaccines and antibodies targeting conserved viral regions has gained increasing attention.

Current vaccine strategies against PDCoV primarily include inactivated vaccines, live-attenuated vaccines, subunit vaccines, viral vector-based vaccines, and nucleic acid-based vaccines [3,64,79]. These approaches have been comprehensively summarized in previous studies and are not discussed in detail here. Looking forward, vaccine development is expected to progressively shift toward parallel strategies that emphasize both cross-protection and broad-spectrum efficacy [79]. Concurrently, the landscape of PDCoV-specific antibody research is expanding rapidly, yielding multiple candidates with promising in vitro and in vivo efficacy. Here, we summarize and analyze representative antibodies, establishing a theoretical basis for future mechanistic studies and potential applications (Figure 4a,b, Table 1 and Table 2).

### 5.1. Antibodies Targeting the S Protein

The S protein of coronaviruses plays a critical role in mediating viral entry, determining host range, and regulating tissue tropism [80]. It also serves as the principal antigen responsible for inducing host immune responses, thus representing a central target for antibody-based studies.

Antisera against the S1, S1A and S1B subunits, obtained from immunized BALB/c mice, effectively block the interaction between pAPN and the PDCoV S protein [8]. Moreover, antisera targeting S1A significantly inhibit the hemagglutination activity of PDCoV toward porcine, avian, and human erythrocytes, suggesting that antibodies directed against S1A may neutralize PDCoV infection by interfering with its sialic acid-binding activity [8]. Subcutaneous immunization of mice with purified S1-CTD protein yielded three PDCoV-specific mAbs: 4E-3, 2A-12, and 4A-8 [81]. Among them, 4E-3 exhibited potent neutralizing activity, with an IC_50_ value of 3.155 μg/mL. Alanine-scanning mutagenesis further identified Asp283, Lys285, and Val288 as critical residues required for 4E-3 recognition [81]. Notably, the epitopes recognized by 4E-3 and 2A-12 are partially or fully exposed on the surface of the S protein and are highly conserved among 25 representative PDCoV strains, indicating that these regions represent ideal immunodominant neutralizing epitopes [82]. Immunization of mice with the 4E3 epitopes peptide (^280^FYSDPKSAV^288^) conjugated to keyhole limpet hemocyanin (KLH) elicited specific neutralizing antibodies [81], highlighting the potential utility of this epitope in vaccine design and therapeutic antibody development. In addition, a newly identified murine mAb, 4A6, demonstrated neutralizing activity against multiple PDCoV strains [83]. The 4A6 epitope (aa 277–552) overlaps with the S receptor-binding region (aa 277–422), suggesting that 4A6 binding may block viral entry by inhibiting S conformational changes [83]. In vivo experiments demonstrated that administration of 4A6 to PDCoV-infected piglets significantly delayed the onset of diarrhea, reduced disease severity, and decreased viral shedding, suggesting its therapeutic potential against PDCoV infection [83].

In addition to the aforementioned murine-derived antibodies, nanobodies and human antibodies targeting PDCoV have also been reported. Yu et al. [84] screened a nanobody, Nb3, with high affinity and specificity for the PDCoV S via a phage display library approach. Based on Nb3, a competitive enzyme-linked immunosorbent assay (cELISA) was subsequently developed, enabling the rapid and efficient identification and quantification of PDCoV-specific antibodies in serum samples. Du et al. [74] immunized H2L2 Harbour mice with S protein and obtained four human PDCoV-neutralizing antibodies (22C10, 42H3, 46E6, and 67B12) (Figure 4a, Table 1). Subsequent structural analyses indicated that the binding of 22C10 to S1A may induce both local and long-range conformational changes in the S protein, promoting the S1B subdomain toward an open conformation; however, this conformational rearrangement does not facilitate APN binding [74]. In contrast, 67B12 directly competes with APN for binding to the S1B subdomain, and its epitope is relatively conserved among deltacoronaviruses. This feature confers cross-reactivity across avian deltacoronaviruses and limits viral escape via mutation, as such mutations would compromise viral fitness [74]. Instead, viral escape is more likely to occur indirectly through allosteric effects at distal sites, thereby highlighting the potential antiviral application of 67B12 [74]. The epitopes of the remaining two antibodies are buried within the prefusion closed conformation of the S trimer and are not affected by glycosylation sites that interfere with APN binding. Their neutralizing activity is therefore likely mediated through the induction of RBD allosteric changes and the reduction in receptor-binding efficiency. Certain antigenic epitopes on the S protein may be spatially masked by glycosylation modifications, which may further enhance the immune evasion capacity of PDCoV. Using these neutralizing antibodies in selection pressure experiments with live virus, researchers simulated the viral evolutionary process and observed that the virus can escape immune recognition by altering the antibodies’ direct binding sites [74]. These findings provide an important theoretical foundation for subsequent vaccine optimization, the development of broadly neutralizing antibodies, and the surveillance of PDCoV variants (Figure 4c).

Alternating administration of the RBD and the S trimer has led to the identification of four mAbs—PD17, PD20, PD33, and PD41—that effectively neutralize vesicular stomatitis virus (VSV)-based PDCoV pseudoviruses [85]. Notably, PD33 and PD41 exhibited potent and broad-spectrum neutralizing activity against multiple emerging human- and animal-derived deltacoronaviruses, as well as a wide range of PDCoV variants [85] (Table 1). Cryo-EM structures and functional assays revealed PD33 and PD41 broadly neutralize PDCoV by competitively binding the RBD from distinct angles, thereby interfering with APN engagement [85]. Importantly, the epitopes recognized by these antibodies are located within highly conserved regions of the RBD. Subsequent deep mutational scanning-based escape mapping further corroborated these findings, demonstrating that several escape-associated hotspot residues are predominantly involved in the interaction between the S protein and the APN receptor [85]. Collectively, these results suggest that the combined use of these two mAbs may more effectively constrain the emergence of viral escape mutants. This observation further implies that combination strategies employing broadly neutralizing antibodies can moderately enhance the barrier against viral immune evasion.

Furthermore, specific antibodies targeting the S2 subunit and other regions of the S protein have also been progressively developed (Figure 4a,b, Table 1). Song et al. [86] employed hybridoma technology to screen two high-affinity mAbs, 2E9 and 11E5, directed against the PDCoV S2 subunit, with affinities of 4.146 nM and 2.133 nM, respectively. The epitopes of both antibodies are surface-exposed on the S protein and are relatively conserved across different PDCoV strains. A sandwich ELISA based on 2E9 and 11E5 was subsequently established, providing a valuable tool for PDCoV detection and S protein detailed structural and functional studies. Additionally, Wang et al. [87] obtained two potent neutralizing mAb, 1C2 and 5H5, which may inhibit the interaction between the S protein and the cellular receptor by recognizing conformational epitopes spanning the S1A and S1B subdomains. Given the epitope characteristics of these antibodies, further development of antibodies targeting the S1 N-terminal domain (S1-NTD), the S2 subunit, and other regions of the S protein remains necessary.

### 5.2. Antibodies Targeting the N Protein

Although antibodies targeting the S protein can effectively neutralize the virus, the broad-spectrum activity and long-term durability of the protective immune responses induced by these antibodies remain limited due to the high genetic variability of the S protein and the low spatial accessibility of certain epitopes. The N protein plays a critical role in viral transcription and replication [88,89]. Moreover, its amino acid sequence is highly conserved and it possesses strong immunogenicity, rendering it an important target for viral diagnosis [15,90]. Consequently, the screening and identification of PDCoV-specific antibodies targeting the N protein have emerged as a key strategy in virological diagnostics (Table 2).

In 2020, Fu et al. [90] immunized mice with three partially overlapping truncated fragments of the N protein (N1, N2, and N3). Using hybridoma technology, they generated and screened five IgG1-type mAbs (3B5, 4E88, 6A5, 7D2, and 11E). Subsequent experiments confirmed that all five antibodies could specifically recognize the N3 truncated fragment. Among these antibodies, 4E88 showed the highest binding affinity to the PDCoV N protein. Homology analysis revealed that this epitope sequence is highly conserved across the 25 selected PDCoV strains. However, its sequence similarity to other prevalent porcine coronaviruses, including PEDV, TGEV, PRCV, SADS-CoV, and PHEV, is less than 22.2%.

Interestingly, Ding et al. [91] used the previously identified N protein-specific mAb 2G12 to perform immunofluorescence assay (IFA) and Western blotting (WB) analyses on a panel of N protein mutants and truncated fragments. Their results confirmed that aa 295–318 within the N-CTD are critical for its nuclear localization. Based on these findings, it is hypothesized that antibodies bearing epitopes near this region may inhibit viral transcription and replication by interfering with the nuclear import of the N protein. However, the exact mechanism remains to be further elucidated. Using similar strategies, Ren et al. [92] obtained two additional highly specific mAbs against the N protein, 6B7 and 7F8, which both recognized the N protein of Asian leopard cat coronavirus. It may suggest a close evolutionary relationship between the two viruses.

Furthermore, He et al. [93] immunized mice with recombinant N protein and generated three mAbs of the IgG2a/κ isotype (3B4, 4D3, and 4E3) via hybridoma technology. Among these, 4D3 showed the highest binding affinity, and the epitope it recognizes (^28^QFRGNGVPLNSAIKPVE^44^) was found to be highly conserved across diverse PDCoV strains. Investigation of another mAb 9G1 of PDCoV N protein [94] found that its epitope sequence (^59^GTPIPPSYAFYY^70^) is highly conserved across the 20 representative PDCoV strains examined but shares low sequence homology with other porcine coronaviruses and with 20 pan-lineage coronavirus strains belonging to the α, β, γ, and δ genera. Notably, only four amino acid residues (G59, F68, Y69 and Y70) within this epitope are relatively conserved across coronaviruses.

It is noteworthy that the N protein epitopes recognized by these antibodies are highly conserved across diverse PDCoV strains and exhibit significant sequence divergence from other prevalent porcine coronaviruses. Consequently, the N protein and its specific antibodies are well suited for the detection and differentiation of PDCoV from other porcine coronaviruses.

### 5.3. Antibodies Targeting Other PDCoV Proteins

In recent years, apart from antibodies targeting the S and N proteins, research on highly specific antibodies against other structural and non-structural proteins of PDCoV has been steadily carried out. The epitopes recognized by these antibodies are generally well conserved and typically show low cross-reactivity with other coronaviruses, suggesting that these antigenic determinants may play vital roles in viral structure, function, and evolution. The identification and characterization of these antibodies also provide novel tools and insights for clarifying the specific functions of corresponding proteins in the PDCoV life cycle and pathogenic mechanisms.

Studies on other coronaviruses have demonstrated that the M protein is the most abundant component of the viral envelope, is relatively well conserved across different strains, and plays an essential role in viral assembly and morphogenesis [13,14,95,96]. Therefore, the development of high-specificity mAbs against the PDCoV M protein may not only facilitate viral detection and differential diagnosis but also provide valuable tools for exploring the assembly mechanism of PDCoV and virus–host interactions. Wu et al. [97] generated an M protein-specific mAb designated 24-A6 via hybridoma technology and confirmed that this antibody exhibits no cross-reactivity with other porcine coronaviruses. Sequence alignment of 240 PDCoV M protein sequences revealed that the epitope recognized by 24-A6 (^103^SPESRL^108^) is highly conserved across different strains. Notably, this epitope resides within the conserved domain SWWSFNPETNNL of the M protein, suggesting that it is potentially associated with the functionally conserved regions of the M protein. Consequently, the 24-A6 epitope can serve as a candidate conserved antigenic region of PDCoV and a broad-spectrum diagnostic target.

**Table 1:** Representative PDCoV-specific neutralizing antibodies and their associated epitopes, neutralizing activities, and applications.

Antibody	Origin/Type	Target	Target Region/Epitope	Primary Application	Neutralizing Activity	Assay System	Cross-Reactivity/Neutralizing Breadth	In Vivo Protective Effects	Reference
Anti-S1/S1A/S1B antisera	Mouse polyclonal sera	S	S1/S1A/S1B	Neutralization	NT titer ≈ 400	Microneutralization assay (CPE-based, live virus)	—	—	[8]
4E-3	Mouse mAb	S	S1-CTD: ^280^FYSDPKSAV^288^ (D283, K285, V288)	Neutralization/Therapy	IC_50_ = 3.155 μg/mL	PRNT (live virus)	—	KLH-S^280–288^ induced partial protection in mouse and pigs	[81]
4A6	Mouse mAb	S	S1: aa 277–552 (S461, P462, T463, E465, Y467)	Neutralization/Therapy	pre-attachment IC_50_ = 0.537 μg/mL; post-attachment IC_50_ = 8.487 μg/mL	PRNT (live virus)	Neutralizing CHN-SC2015, SCNC201705 and CHN-SCMY2021-01 (PRNT, live virus)	Partial protection in pigs	[83]
22C10	Human mAb	S	S1A (L45, Y46, S231)	Neutralization/Research	IC_50_ = 1.27 μg/mL	Microneutralization assay (IFA-based, live virus)	No cross-reactivity with tested delta-CoVs or alpha-CoVs	—	[74]
42H3/46E6	Human mAbs	S	S1B (42H3: S362, E410; 46E6: S362)	Neutralization/Research	IC_50_ = 0.15 μg/ML; IC_50_ = 0.08 μg/mL	Microneutralization assay (IFA-based, live virus)	Cross-reactivity with coronaviruses HKU11-934 and ISU42824 (S1A, ELISA)	—	[74]
67B12	Human mAb	S	S1B (W396)	Neutralization/Therapy	IC_50_ = 0.03 μg/mL	Microneutralization assay (IFA-based, live virus)	Cross-reactivity with HKU11, ISU73347 and ISU42824 (S1A, ELISA)	—	[74]
PD33	Human mAb	S	S1-CTD (RBD): (F318, Y394, W396, Y398, R401)	Neutralization/Therapy	IC_50_ < 0.5 μg/mL	Microneutralization assay (Pseudovirus, VSV-PDCoV)	—	—	[85]
PD41	Human mAb	S	S1-CTD (RBD): (H315, D317, F318, E320, D324, Y394)	Neutralization/Therapy	IC_50_ < 0.5 μg/mL	Microneutralization assay (Pseudovirus, VSV-PDCoV)	Broadly Neutralize 6 PDCoV variants: USA_IL121_2014, Thai_S5011_2015, HKD-JPN_2016, HKU15_SD2018, 110_1197_TW, and Hu-PDCoV isolate (Pseudovirus, VSV-PDCoV)	—	[85]
PD17/PD20	Human mAbs	S	S1-CTD (RBD), Epitope near the RBD equator that partially overlaps receptor-binding loops	Neutralization	IC_50_ ≈ 5 μg/mL	Microneutralization assay (Pseudovirus, VSV-PDCoV)	—	—	[85]
1C2/5H5	Mouse mAbs	S	S1A-S1B inter-domain linker	Neutralization/Therapy	NT titer > 1:16,384	Microneutralization assay (IFA-based, live virus)	—	—	[87]

Residues in parentheses indicate the key residues within the epitope in the “Target region/epitope” column. The IC_50_ values of PD17/PD20/PD33/PD41 were not reported as exact numbers in the original paper; the approximate values shown here were estimated from the neutralization curves in Figure S1 of that study [85]. It should be noticed that pseudovirus-based results may not fully recapitulate neutralization against authentic live PDCoV. The “—” indicates that no associated data was reported in the corresponding reference. Domain boundaries follow the corresponding original references, variations in domain boundaries may arise from different reference strains and non-standardized domain demarcation. Abbreviation: NT, neutralizing titers; PRNT, Plaque-reduction neutralization test.

**Table 2:** Diagnostic and research antibodies targeting PDCoV structural and accessory proteins.

Antibody	Origin/Type	Target	Target Region/Epitope	Primary Application	Cross-Reactivity	Reference
2A-12	Mouse mAb	S	S1-CTD: ^506^TENNRFTT^513^	Research	—	[81]
4A-8	Mouse mAb	S	S1-CTD: Conformational epitope aa 590–641	Research	—	[81]
Nb3	Alpaca, Nanobody	S	—	Diagnosis	No cross-reaction with positive sera of PEDV, TGEV, PoRV, PRV, CSFV, PRRSV, PCV2	[84]
2E9/11E5	Mouse mAbs	S	S2: 2E9: ^574^PSAF^577^; 11E5: ^605^NSRC^608^	Diagnosis/Research	No cross-reactivity with TGEV, PEDV, PSV, or PAstV-5	[86]
4E88	Mouse mAb	N	N3 fragment: ^309^KPKQQKKPK^317^	Diagnosis/Research	No cross-reactivity with PEDV or TGEV	[90]
2G12	Mouse mAb	N	N-CTD aa 295–318	Research	—	[91]
6B7/7F8	Mouse mAbs	N	6B7: aa 326–333; 7F8: aa 251–276	Diagnosis/Research	Cross-reacts with Asian leopard cat coronavirus N protein	[92]
4D3	Mouse mAb	N	^28^QFRGNGVPLNSAIKPVE^44^	Diagnosis/Research	No cross-reactivity with PEDV or TGEV	[93]
9G1	Mouse mAb	N	^59^GTPIPPSYAFYY^70^	Diagnosis/Research	—	[94]
24-A6	Mouse mAb	M	^103^SPESRL^108^	Diagnosis/Research	No cross-reactivity with SADS-CoV, PEDV, GETV, SVA or PSV	[97]
5-A11	Mouse mAb	NS6	^70^EYGSIYGKDFI^80^	Diagnosis/Research	No cross-reactivity with PEDV, SVA GETV, PSV, or SADS-CoV	[98]
3D5	Mouse mAb	NS6	^25^VPELIDPLVK^34^	Diagnosis/Research	No cross-reactivity with TGEV, or PEDV	[99]

The “—” indicates that no associated data was reported in the corresponding reference.

Given its essential roles in the viral life cycle and high sequence conservation, NS6 represents a promising antigenic target. Wu et al. [98] screened a PDCoV-NS6 specific mAb termed 5-A11, which showed no cross-reactivity with several prevalent porcine viruses. The epitope recognized by 5-A11 (^70^EYGSIYGKDFI^80^) is highly conserved across 202 PDCoV isolates, with approximately 96.5% sequence identity. However, obvious sequence variations were detected among 17 examined deltacoronavirus isolates, indicating favorable species specificity of this antibody. In addition, Liu et al. [99] identified another NS6-specific mAb, 3D5 (IgG2b), which targets a relatively conserved linear epitope (^25^VPELIDPLVK^34^). Collectively, these results further confirm that NS6 contains multiple conserved antigenic regions with immunological relevance.

In 2017, Fang et al. [35] immunized mice with bioinformatically predicted and recombinantly expressed NS7 protein and successfully obtained four NS7-reactive mAbs, designated 1C8, 2E11, 3G10, and 1A10. Using these antibodies, the researchers further identified a novel accessory protein encoded by PDCoV, termed NS7a. While NS7a exerts crucial functions in viral immune regulation and represents a promising target for antiviral therapy, studies on its antigenic structural features remain scarce.

## 6. Discussion and Future Perspectives

As an emerging enteric pathogen, PDCoV’s biological characteristics, broad host range, and immune evasion mechanisms collectively form the basis of its pathogenicity. Global surveillance and interdisciplinary research are crucial for controlling its spread. Future research should focus on cross-species transmission mechanisms, vaccine development, and long-lasting mucosal immune protection to address potential public health challenges.

The broad host range of PDCoV supports the urgent need for a “One Health” approach. While swine are the primary agricultural hosts, the widespread detection of PDCoV in diverse avian species and mammals based primarily on experimental susceptibility points to a potential complex ecological reservoir; however, for many species this evidence rests primarily on experimental susceptibility and does not confirm a natural reservoir or intermediate host role. Notably, the identification of independent zoonotic spillover events in Haitian children provides evidence that the virus can cross the species barrier and infect humans, although definitive evidence of sustained human infection and transmission remains insufficient. Future epidemiological research must shift from reactive outbreak reporting to proactive, active surveillance at the human–animal interface, particularly in high-risk environments such as live poultry markets, swine farms, and rural communities. Furthermore, while APN/CD13 is the best-characterized functional receptor facilitating viral entry, the residual susceptibility of APN-knockout pigs strongly suggests the existence of alternative co-receptors or entry factors. Identifying these unknown host factors is a critical next step for fully elucidating the molecular determinants of PDCoV’s broad host tropism and its interspecies transmission trajectory. The prevention and control of PDCoV transmission and epidemics urgently call for the development of more potent antiviral strategies.

A comprehensive understanding of diverse PDCoV antigenic targets and the functional properties of their cognate antibodies is essential for clarifying viral infection mechanisms, evaluating cross-species transmission risks, and developing broad-spectrum antiviral agents. To date, studies on PDCoV antibodies have mainly focused on diagnostic antibodies, which are predominantly of murine origin and belong to the IgG isotype [90,93,94], while research on antibodies from other sources and isotypes remains relatively scarce. Although studies on human neutralizing antibodies against PDCoV have increased in recent years [74,85], the functional profiles and action mechanisms of most such antibodies have not been fully elucidated. Furthermore, investigations using porcine and other animal models of PDCoV infection have revealed that PDCoV primarily induces gastrointestinal lesions and tissue damage [60,61,100]. Nevertheless, research on PDCoV mucosal immunity and IgA antibodies is still limited. Accordingly, in-depth research and development of IgA antibodies may serve as a promising alternative for PDCoV antibody-based therapy. Innovative delivery platforms—such as oral/enteric-coated nanoparticle vaccines, adenovirus-vectored mucosal vaccines, or mRNA lipid nanoparticles targeted to the gut-associated lymphoid tissue—represent the frontier of PDCoV vaccinology.

Beyond the S protein, research on other PDCoV antigenic targets is still inadequate. Proteins such as NS6, NS7a, and the E protein viroporin play indispensable roles in innate immune antagonism and modulating host apoptosis/autophagy. Developing small-molecule inhibitors or specifically designed mAbs that target these non-structural and accessory components could effectively disarm the virus’s immune evasion machinery, restoring the host’s natural antiviral state.

In summary, PDCoV represents a compelling model for studying coronavirus evolution, cross-species transmission, and mucosal immunology. Key knowledge gaps remain in understanding its non-APN receptors, the structural dynamics of its S protein, and the correlates of protective immunity in the gut. Proactive investment in PDCoV research will not only protect the swine industry but also enhance pandemic preparedness against emerging deltacoronaviruses with zoonotic potential.

## Figures and Tables

**Figure 1:**
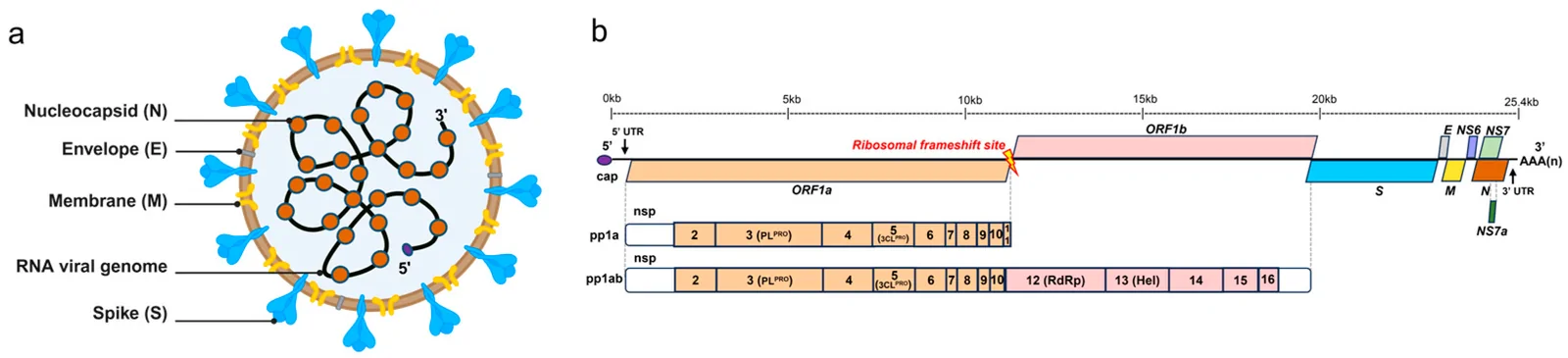
Schematic illustration of PDCoV virion structure and genomic architecture. (**a**) Schematic diagram of the PDCoV virion, highlighting the major viral structural proteins. (**b**) Genomic organization of PDCoV, referenced to the porcine deltacoronavirus strain HKU15-44 (GenBank accession: JQ065042.2). Naturally occurring genomic variations are present among different PDCoV isolates. The open reading frames (ORFs) and corresponding color coding are displayed as follows: *ORF1a* (orange), *ORF1b* (pink), *S* gene (blue), *E* gene (gray), *M* gene (yellow), *NS6* gene (purple), *N* gene (reddish-brown), *NS7* gene (light green), and *NS7a* gene (dark green). Slanted quadrilaterals represent the genes, and rounded squares represent the putative encoded proteins. This unified color scheme is applied consistently across all figures in this article. Papain-like protease, PL^pro^; chymotrypsin-like protease, 3CL^pro^; RNA-dependent RNA polymerase, RdRp. https://BioRender.com/4jbi9kl, accessed on 15 September 2026.

**Figure 3:**
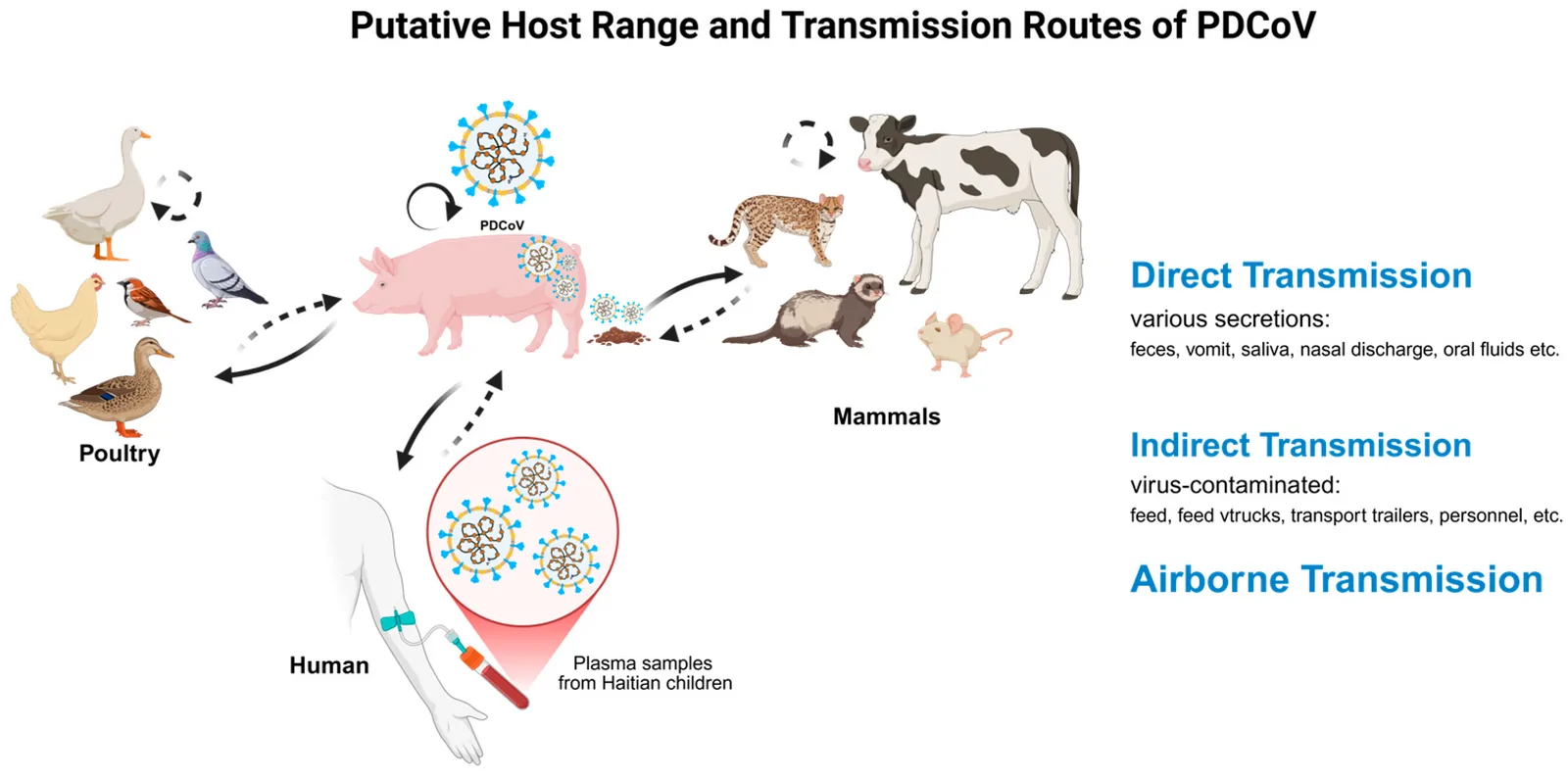
Schematic illustration of putative PDCoV host range and transmission routes. PDCoV infects both poultry and mammalian hosts, primarily through three possible epidemiological routes: direct contact, indirect fomite-mediated, and airborne aerosol transmission. Solid arrows denote transmission events confirmed experimentally in animal models, at the cellular level, or supported by epidemiological evidence; dashed arrows indicate potential or inferred transmission routes that have not been definitively documented but cannot be excluded. https://BioRender.com/rgu32tu, accessed on 15 September 2026.

**Figure 4:**
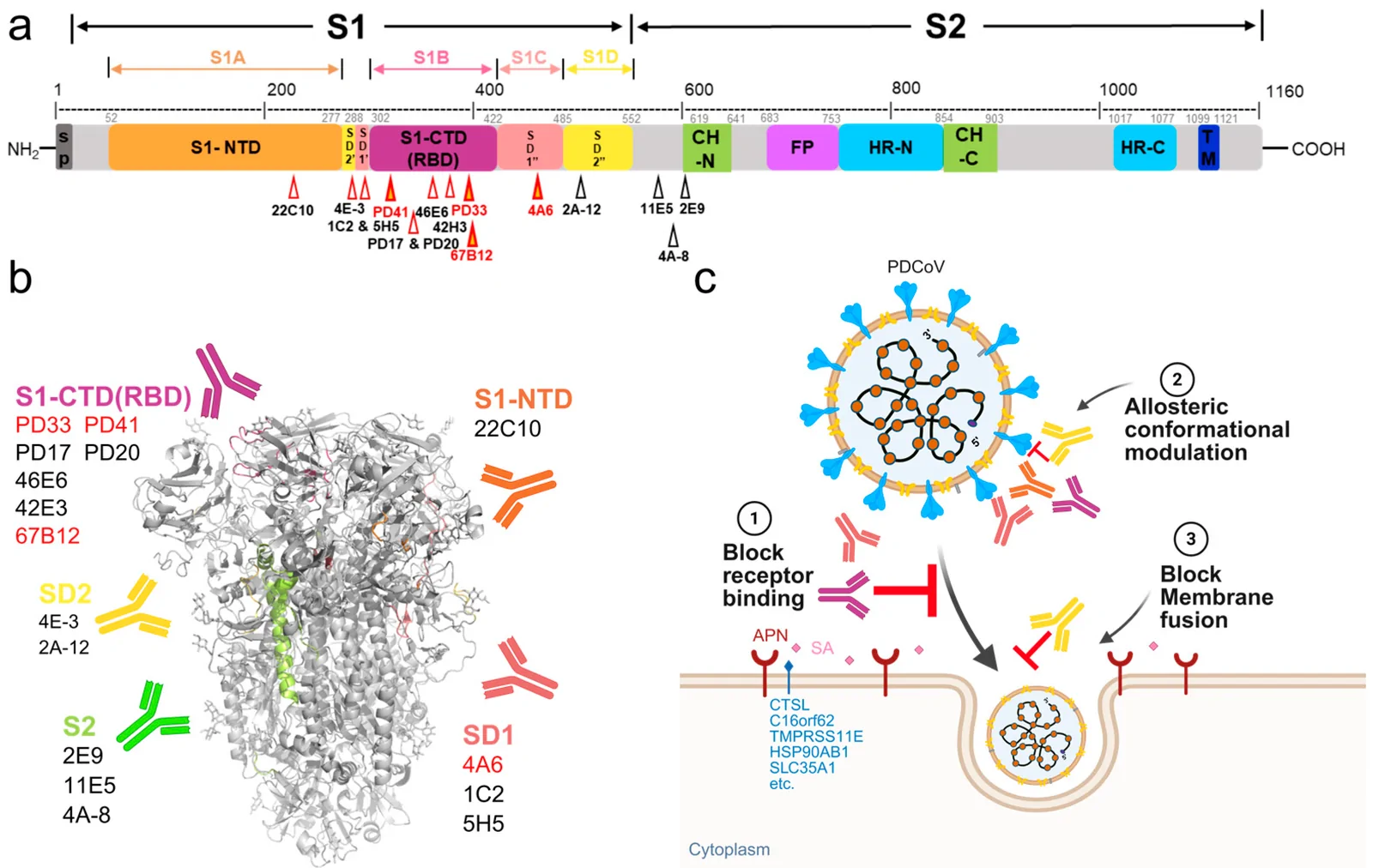
Structural features of the PDCoV S protein, spatial distribution of representative antibody epitopes, and proposed mechanisms of antibody-mediated neutralization. (**a**) Schematic domain organization of the full-length PDCoV S protein. The diagram is based on the reference strain HKU15 (NCBI Taxonomy ID: 1586324). Domain boundaries and amino acid positions are annotated according to this strain. Owing to genetic variations among circulating PDCoV isolates, minor discrepancies in domain boundaries and epitope locations may exist. The S1 subunit may be further divided into S1A, S1B, S1C, and S1D subdomains, although no unified nomenclature exists. Triangles indicate the approximate locations of antibody epitopes on the S protein. Red triangles represent neutralizing antibodies; filled red triangles represent broadly neutralizing antibodies; open black triangles represent non-neutralizing antibodies. SP, signal peptide; S1-NTD, N-terminal domain of the S1 subunit; S1-CTD, C-terminal domain of the S1 subunit; RBD, receptor-binding domain; SD1 (SD1′/SD1″) and SD2 (SD2′/SD2″), subdomains; CH-N, N-terminal central helix; CH-C, C-terminal central helix; FP, fusion peptide; HR-N, N-terminal heptad repeat; HR-C, C-terminal heptad repeat; TM, transmembrane. (**b**) Spatial distribution of representative antibody epitopes on the S protein. The three-dimensional structure of the S protein was derived from PDB entry 6b7n, illustrating the relative positions of epitopes targeted by selected antibodies. (**c**) Proposed mechanisms of antibody-mediated neutralization. Antibodies neutralize PDCoV through three proposed mechanisms based on published reports: (i) blocking the interaction between the S protein and its host receptor; (ii) inducing conformational changes in the S protein that impair its functional transition; and (iii) directly inhibiting S2 subunit-mediated membrane fusion. Black arrows indicate the proposed direction of neutralization; red blocking symbols denote inhibition of receptor binding or membrane fusion. https://BioRender.com/uv48zbs, accessed on 15 September 2026.

## Data Availability

No new data were created or analyzed in this study. Data sharing is not applicable to this article.

## Abbreviations

The following abbreviations are used in this manuscript:

APN	Aminopeptidase N
C16orf62	Chromosome 16 Open Reading Frame 62
CTD	C Terminal Domain
CTSL	Cathepsin L
DMVs	Double Membrane Vesicles
dpi	Days Post Infection
E	Envelope Protein
ER	Endoplasmic Reticulum
ERGIC	ER Golgi Intermediate Compartment
GN	Gnotobiotic
HSP90AB1	Heat Shock Protein 90 Alpha Family Class B Member 1
IFN	Interferon
M	Membrane Protein
mAbs	Monoclonal Antibodies
N	Nucleocapsid Protein
NA	Neuraminidase
Nsp	Nonstructural Proteins
NS6	Nonstructural Protein 6
NTD	N Terminal Domain
ORFs	Open Reading Frames
PDCoV	Porcine Deltacoronavirus
PEDV	Porcine Epidemic Diarrhea Virus
RBD	Receptor Binding Domain
rER	Rough Endoplasmic Reticulum
RNP	Ribonucleoprotein
RTC	Replication Transcription Complex
S	Spike Protein
SA	Sialic Acid
S1 CTD	C Terminal Domain of the S1 Subunit
Sg mRNAs	Subgenomic mRNAs
SLC35A1	Solute Carrier Family 35 Member A1
TGEV	Transmissible Gastroenteritis Virus
TMD	Transmembrane Domain
TMPRSS11E	Transmembrane Serine Protease 11E
UPR	Unfolded Protein Response
